# *BCAR3* Hypomethylation as a Potential Diagnostic Marker for Thyroid Cancer and Its Mechanism via Promoting EMT and AKT/mTOR Pathway

**DOI:** 10.3390/cancers18020267

**Published:** 2026-01-15

**Authors:** Wenkang Yu, Yizhu Mao, Yifei Yin, Jiacheng Yang, Yi Zhang, Xuandong Huang, Yifen Zhang, Chenxia Jiang, Rongxi Yang

**Affiliations:** 1 Department of Epidemiology, School of Public Health, Nanjing Medical University, Nanjing 211166, China; 2 Hongkou District Center for Disease Control and Prevention, Shanghai (Hongkou District Institute of Health Supervision, Shanghai), Shanghai 200434, China; 3 Department of Thyroid and Breast Surgery, The Affiliated Huai’an Hospital of Xuzhou Medical University and The Second People’s Hospital of Huai’an, Huai’an 223002, China; 4 Department of Pathology, Jiangsu Province Hospital of Chinese Medicine, Affiliated Hospital of Nanjing University of Chinese Medicine, Nanjing 210029, China; 5 Department of Pathology, Affiliated Hospital of Nantong University, Nantong 226001, China

**Keywords:** *BCAR3*, DNA methylation, biomarker, thyroid cancer, EMT

## Abstract

Background: *BCAR3* has been implicated in various cancers, yet its role in thyroid cancer (TC) remains unclear. This study aimed to investigate the methylation status, functional effects, and underlying mechanisms of *BCAR3* in TC. Methods: *BCAR3* methylation was analyzed using matrix-assisted laser desorption/ionization–time-of-flight (MALDI-TOF) mass spectrometry in 422 TC and 371 benign thyroid nodule samples. Expression levels were assessed via immunohistochemistry, qPCR, and Western blot. Functional assays including proliferation, migration, and invasion were performed after *BCAR3* knockdown. Rescue experiments using a PI3K activator were conducted to examine pathway mechanisms. Results: *BCAR3* was significantly hypomethylated in TC compared to benign tissues (*p* < 0.001), with CpG_6 most strongly associated with TC risk (odds ratio, OR = 1.73, *p* < 0.001). Notably, *BCAR3* hypomethylation was more pronounced in cases with larger tumor size and advanced disease stage. Furthermore, *BCAR3* methylation showed differential patterns across TC subtypes, with medullary thyroid carcinoma exhibiting the lowest methylation levels. BCAR3 expression was upregulated in TC tissues and cell lines (*p* < 0.05). Mechanistically, *BCAR3* knockdown reduced phosphorylation of AKT/mTOR and altered expression of epithelial-to-mesenchymal transition (EMT) marker, characterized by an increase in E-cadherin and decreases in Vimentin and N-cadherin, and consequently suppressed proliferation, migration, and invasion (*p* < 0.05). Rescue experiments with a PI3K activator showed a trend towards restoration of these effects, although not to the level of the control groups. Conclusions: *BCAR3* hypomethylation contributes to TC cells’ proliferation, migration, and invasion by promoting AKT/mTOR activation and EMT. These findings highlight the potential of *BCAR3* methylation as both a biomarker and a therapeutic target in TC.

## Simple Summary

Distinguishing malignant from benign thyroid nodules preoperatively remains a significant diagnostic challenge. This study investigated the role of a gene called *BCAR3* in thyroid cancer. Using a large cohort of 793 clinical samples and cell experiments, we discovered that *BCAR3* loses a specific regulatory mark (DNA methylation) in thyroid tumors, and we demonstrated that *BCAR3* promotes tumor cell growth, migration, and invasion through the AKT/mTOR pathway and epithelial–mesenchymal transition. Therefore, measuring *BCAR3* methylation has the potential to serve as a novel diagnostic biomarker, and *BCAR3* itself represents a promising future therapeutic target for thyroid cancer.

## 1. Introduction

Thyroid cancer (TC) represents the most common endocrine malignancy [1]. Its global incidence has risen steadily over the past few decades [2,3], Recent epidemiology shows that its prevalence in China has eclipsed that of many Western countries, making it a significant public health concern [4,5]. This trend establishes TC as a considerable public health issue and underscores the growing challenges in clinical decision-making [6].

Fine-needle aspiration biopsy (FNAB) serves as the primary diagnostic tool for the definitive evaluation of thyroid nodules [7]. While FNAB is a minimally invasive and highly effective method for distinguishing between benign and malignant nodules, it is not without limitations. The diagnostic accuracy of FNAB may be compromised by indeterminate results, which occur in 20–30% of cases and are classified within the Bethesda system as categories III–IV [8]. These indeterminate results can lead to diagnostic uncertainty, requiring additional invasive procedures or even surgery, thereby increasing patient anxiety and healthcare costs [9]. To address these limitations, molecular testing has emerged as a valuable adjunct, enabling more precise differentiation between benign and malignant thyroid nodules through the analysis of mutations in genes. Yet these molecular markers have inherent limitations: lack sufficient sensitivity (*BRAF*) [10], compromise specificity due to presence in both benign and malignant lesions (*RAS*) [11], or too rare for routine diagnostic use (*TERT*) [12]. Therefore, there is an urgent clinical need for more robust and standardized diagnostic biomarkers. The identification of such markers is crucial not only for accurate diagnosis but also for guiding personalized therapeutic strategies [13,14].

DNA methylation is a fundamental and widespread epigenetic mechanism that regulates gene expression in living organisms [15]. Numerous studies have confirmed that changes in DNA methylation are early and frequent events in the development of cancer [16]. The stability of the DNA methylation process allows for quantitative analysis, with simple sample processing, reliable detection methods, low detection dosage, and excellent reproducibility [17]. In TC, both global hypomethylation and site-specific hypermethylation events have been observed, indicating the significant role of epigenetic alterations in this malignancy [18]. Given the critical role of DNA methylation in gene regulation and its early involvement in carcinogenesis, methylation markers are emerging as promising diagnostic and prognostic tools, offering valuable insights into tumor behavior and patient outcomes in TC.

The *BCAR3* (Breast Cancer Anti-Estrogen Resistance 3) gene was initially identified in breast cancer, where it plays a role in anti-estrogen resistance [19]. The BCAR3 protein contains an N-terminal Src-homology 2 (SH2)-like domain, which enables it to interact with tyrosine-phosphorylated residues on various signaling proteins, including Src family kinases [20,21]. Through interaction with p130Cas (BCAR1), a key adaptor protein involved in focal adhesion dynamics, BCAR3 facilitating tumor cell migration, invasion, and adhesion disassembly [22,23,24,25]. Additionally, BCAR3 has been implicated in the activation of the AKT/mTOR signaling pathway, promoting cancer cell proliferation and survival [26]. Notably, emerging evidence suggests that BCAR3 may promote epithelial–mesenchymal transition (EMT), a critical process in cancer metastasis [27,28]. Recent studies have reported aberrant BCAR3 expression in multiple malignancies, suggesting its potential role in oncogenesis [29,30]. Our pilot study on 7 TC and 11 benign thyroid nodules (BTNs) using Illumina 850K assay and RNA-seq showed differential methylation and expression in *BCAR3* (Figure S1), which was not reported in TC previously. Here we investigate the methylation status of *BCAR3* in thyroid tumors in a substantial clinical cohort and evaluate its potential as a diagnostic biomarker, while also exploring the functional consequences of *BCAR3* dysregulation in TC. While DNA methylation alterations frequently contribute to gene dysregulation in cancer, the relationship between *BCAR3* methylation and cancer remains poorly characterized. Here we investigate the methylation status of *BCAR3* in thyroid tumors and evaluate its potential as a diagnostic biomarker, while also exploring the functional consequences of *BCAR3* dysregulation in TC.

## 2. Materials and Methods

### 2.1. Study Design and Patients

This study included a total of 793 patients from three participating medical centers: Huai’an Hospital of Xuzhou Medical University (Center I, 169 BTNs and 136 TC cases), the Affiliated Hospital of Nantong University (Center II, 173 BTNs and 233 TC cases), and Jiangsu Province Hospital of Chinese Medicine (29 BTNs and 65 TC cases). The cohort comprised 371 BTN cases and 422 TC cases. Baseline characteristics of the study participants from Center I and Center II are summarized in Table 1, while the complete characteristics of all included subjects are provided in Table S1. All diagnoses were confirmed by two independent pathologists according to the eighth edition of the American Joint Committee on Cancer (AJCC) Staging System. Formalin-fixed paraffin-embedded (FFPE) samples were collected prior to any treatment. This retrospective study was conducted in accordance with the Declaration of Helsinki and was approved by the Institutional Review Boards of Nanjing Medical University (protocol code [2020] 528) and the Medical Ethics Committee of Huai’an Second People’s Hospital (protocol code HEYLL202315). Informed consent was obtained from all participants.

### 2.2. Methylation Analysis by MALDI-TOF Mass Spectrometry

The CpG sites within the *BCAR3* gene were selected based on two criteria: (1) annotation in the Genome Browser (University of California, Santa Cruz (UCSC), build GRCh38/hg38); and (2) location within the predicted promoter-like signature region. The CpG site designated as cg10546447 was selected for further investigation. Subsequently, an amplicon of 140 base pairs and within the promoter region was designed to encompass cg10546447 (referred to as CpG_2 in the amplicon) and five quantifiable flanking CpG sites for mass spectrometry analyses (Table S2). CpG_2.3.4.5 represents the four CpG sites located within the same fragment, and their methylation levels are presented as an average. Schematic of the *BCAR3* amplicon for methylation analysis is shown in Figure S2.

DNA methylation levels were assessed by semiquantitative analysis using the Agena MassARRAY system (Agena Bioscience, San Diego, CA, USA), which is based on MALDI-TOF mass spectrometry, following a previously established protocol [31]. Briefly, PCR amplification was carried out using bisulfite-converted DNA, followed by shrimp alkaline phosphatase treatment and T-cleavage reaction. The products were dispensed onto a 384 SpectroCHIP and analyzed using a MassARRAY system (Agena Bioscience). Methylation ratios were generated using EpiTyper v1.3 software. The reliability and accuracy of the matrix-assisted laser desorption/ionization–time-of-flight (MALDI-TOF) MS platform (EpiTyper) for DNA methylation quantification have been demonstrated in a large-scale benchmarking study, which showed it performs with high accuracy and excellent concordance with bisulfite sequencing and pyrosequencing [32]. In addition, our MALDI-TOF MS data was replicated by Illumina 850K (Illumina, San Diego, CA, USA) showing similar methylation levels with the same 7 TC and 11 BTN samples (Figure S3).

### 2.3. Immunohistochemistry (IHC)

FFPE sections (4-μm) were deparaffinized, rehydrated, and subject to antigen-retrieved in citrate buffer (pH 6.0). After peroxidase blocking with 3% H_2_O_2_, sections were incubated with 5% BSA and then with anti-BCAR3 antibody (1:100, Abmart, TD3048, Shanghai, China) at 4 °C overnight. Detection was performed with an HRP-conjugated secondary antibody, followed by DAB development and hematoxylin counterstaining. Protein expression was quantified using a color deconvolution algorithm in ImageJ (Fiji, version 1.54p) to isolate the DAB channel. Two independent pathologists, blinded to the clinical data and working in consensus, first defined the region of interest (ROI) around tumor cells. A consistent intensity threshold for positive signal was pre-determined on a set of training images and applied uniformly to all subsequent analyses. The percentage of positive cells was automatically calculated by the software within each ROI. Inter-observer agreement was assessed across all samples, demonstrating unanimous agreement in 94.4%.

### 2.4. Cell Culture and Transfection

All Human thyroid cell lines (KTC-1, TPC-1, and Nthy-ori3-1) were obtained from the Chinese Academy of Sciences (Shanghai, China), and routinely maintained in complete RPMI 1640 (10% FBS) at 37 °C and 5% CO_2_. Gene silencing was achieved by transfecting cells with *BCAR3*-specific siRNA or a control siRNA using Lipofectamine 3000 (Thermofisher, L3000075, Shanghai, China). The sequences of all siRNA constructs used are provided in Table S3. Knockdown efficiency and phenotypic effects were verified for all three siRNAs. The most efficient siRNA (si*BCAR3*-605, GenePharma, Suzhou, China) was selected for subsequent functional and rescue experiments. For rescue experiments, cells were treated with 10 μM PI3K activator 1,3-dicaffeoylquinic acid (MCE, HY-N1412, Shanghai, China) [33,34].

### 2.5. RNA Extraction and Quantitative PCR

Total RNA was isolated from cells using Trizol extraction kit (Invitrogen, 15596018, Shanghai, China), reverse-transcribed, and quantified using SYBR Green mix (Vazyme, R222-01, Nanjing, China) on an ABI 7500 system. Gene expression levels were normalized to GAPDH as an internal control, and relative quantification was determined via the 2^−ΔΔCT^ method. All primer sequences are provided in Table S4.

### 2.6. Western Blot

Total protein was extracted from cells using RIPA lysis buffer (Beyotime, P0013B, Shanghai, China) supplemented with 1% protease inhibitors (RUIBIO, BP2655, Guangzhou, China). Protein concentration was quantified with a BCA assay kit (Biosharp, BL521A, Hefei, China). Subsequently, equal amounts of protein (25 μg) were electrophoretically resolved on 10% SDS-PAGE gels and transferred onto PVDF membranes (Millipore, IPVH00010, Shanghai, China). The membranes were blocked with 5% non-fat milk and then probed with primary antibodies at 4 °C overnight (anti-BCAR3, 1:1000, Abmart, TD30488, Shanghai, China; anti-GAPDH, 1:10,000, Proteintech, 60004-1-Ig, Wuhan, China; anti-β-actin, 1:5000, Proteintech, 66009-1-Ig; anti-Vimentin, 1:2000, Abclonal, A19607, Wuhan, China; anti E-cadherin, 1:20,000, Proteintech, 20874-1-AP; anti-N-cadherin, 1:1000, Abmart, T55015; anti-AKT, 1:5000, Abclonal, A18675; anti-pAKT, 1:1000, Abclonal, AP1068; anti-mTOR, 1:5000, Proteintech, 66888-1-Ig; anti-pmTOR, 1:2000, Proteintech, 67778-1-Ig), followed by HRP-conjugated secondary antibodies (HRP-Goat Anti-Mouse IgG, 1:5000, Proteintech, RGAM001; HRP-Goat Anti-Rabbit IgG, 1:5000, Proteintech, RGAR001) at 37 °C for 1 h. Signals were detected using ECL reagent (Vazyme, E423), and band densities were quantified using ImageJ software (Fiji, version 1.54p).

### 2.7. Cell Function Assays

#### 2.7.1. Cell Viability Assay

Cell viability was measured daily from Day 0 to Day 5 using the CCK-8 assay in 96 well plates. In Day 0, cells were seed in six 96 well plates: four experimental group in each plate, and each group were replicated in 6 parallel wells with 5 × 10^3^ cells in each well. From Day 0 on, one 96 well plate was measured by the CCK-8 assay every 24 h, till Day 5. For each measurement, 10 μL of CCK-8 solution was added to the well, followed by 3 h’ incubation and the absorbance was measured at 450 nm. The Statistical comparisons between groups at each time point were performed on the raw OD values using unpaired *t*-tests.

#### 2.7.2. Colony Formation Assay

Cells were seeded in 6-well plates (200 cells/well) and cultured for 2 weeks. The resulting colonies were fixed with 4% paraformaldehyde, stained with 0.1% crystal violet, and counted. The cloning efficiency was calculated for each group using the formula: (numbers of colonies/number of cells seeded) × 100%.

#### 2.7.3. Wound Healing Assay

Cells were cultured in 6-well plates until confluent (10% FBS). Then, three parallel scratches were created by pipette tips per well, and the medium for cell culture were changed to low-serum (1% FBS). Wound areas were imaged at 0 and 48 h under a phase-contrast microscope. The cell migration rate was quantified using ImageJ software and calculated as: (Wound Area at 0 h–Wound Area at 48 h)/Wound Area at 0 h × 100%, with normalization to the 0-h time point of each experimental group. Data are presented as the cell migration rate.

#### 2.7.4. Transwell Migration and Invasion Assay

Cell migration and invasion were assessed using 24-well Transwell chambers. For invasion assays, chambers were pre-coated with Matrigel (1:8 dilution); uncoated chambers were used for migration. Cells (3 × 10^5^ in serum-free medium) were seeded into the upper chamber, with complete medium as a chemo-attractant in the lower chamber. After 24 h, cells on the upper surface were removed, and cells that migrated to the lower surface were fixed and stained with 0.1% crystal violet. The number of invaded cells was determined by counting the stained cells in three random microscopic fields. The average count per field for each experimental group was then directly compared for statistical analysis using the unpaired *t*-test.

### 2.8. Immunofluorescence Staining (IF)

Cells were subjected to fixation with 4% paraformaldehyde and permeabilization with 0.1% Triton X-100 (Beyotime, P0096), followed by blocking with 5% FBS. Samples were incubated overnight at 4 °C, with primary antibodies against E-cadherin (1:100, Abcam, Ab40772, Shanghai, China) and Vimentin (1:500, Abclonal, A19607). After washing, the samples were probed with Cy3- or FITC-conjugated secondary antibodies (Cy3–conjugated Affinipure Goat Anti-Rabbit, Proteintech, SA00009-2; FITC–conjugated Affinipure Goat, Proteintech, SA00003-1). Nuclei were counterstained with Hoechst dye (Beyotime, C1017) dye for 15 min at room temperature in the dark. Finally, images were captured using a fluorescence microscope.

### 2.9. Statistical Analyses

Continuous variables are summarized as median with interquartile range (IQR). Group differences in *BCAR3* methylation levels were analyzed using the Mann–Whitney U test for two-group comparisons and the Kruskal–Walli’s test for multi-group comparisons. Where the Kruskal–Walli’s test was significant, post hoc pairwise testing was performed using the Dunn’s test, and *p* values were adjusted by the Bonferroni method (multiplying the raw *p* value by the number of comparisons, n = 3).  All statistical analyses were conducted using SPSS (version 28.0; IBM Corp., Armonk, NY, USA).

## 3. Results

### 3.1. BCAR3 Hypomethylation in TC Cases Compared to BTN Subjects

**Figure 1:**
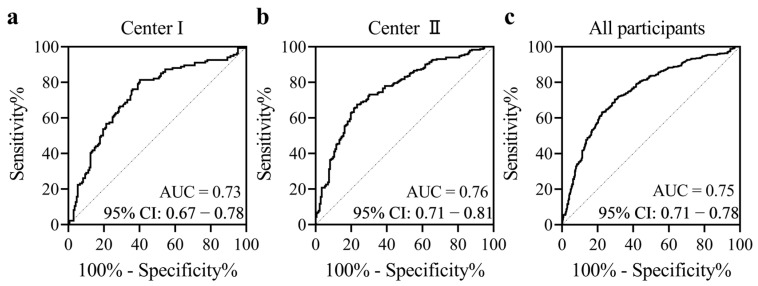
Diagnostic value of *BCAR3* methylation in distinguishing thyroid cancers from benign thyroid nodules. The methylation levels of six CpG sites within the *BCAR3* gene were utilized to generate a prediction probability via logistic regression with age and sex adjusted. (**a**) Center I, (**b**) Center II, (**c**) All participants combined center I and II. Abbreviations: AUC, area under the curve. CI, confidence interval.

We compared the methylation levels between the TC cases and subjects with benign thyroid nodules using a logistic regression model adjusted for age and sex, and found that the methylation levels of all six CpG loci in *BCAR3* amplicon were significantly lower in the TC group than in the BTN group. Specifically, the methylation values for CpG_1, CpG_2.3.4.5, and CpG_6 in TC were 0.65, 0.65, and 0.81, respectively, compared to 0.78, 0.74, and 0.92 in BTN (Table 2). Furthermore, our analysis revealed a significant association between *BCAR3* hypomethylation and TC. After adjusting for age and sex, the odds ratios (ORs) per 10% reduction in methylation for all CpG sites were 1.68 for CpG_1, 1.54 for CpG_2.3.4.5, and 1.73 for CpG_6, indicating an increased risk of TC (95% CI: 1.51–1.86, 1.39–1.70, 1.53–1.96 respectively; all *p* < 0.001 after Bonferroni adjustment; Table 2). This association was consistently observed in both participating centers, with Center I showing ORs ranging from 1.40 to 1.53 and Center II from 1.55 to 1.87 per 10% reduction in methylation (all adjusted *p* < 0.001; Table S5). To evaluate the diagnostic potential of *BCAR3* methylation, we performed ROC analysis. The overall model achieved an area under the curve (AUC) of 0.75 (95% CI: 0.71–0.78), with a sensitivity of 63.2% and a specificity of 59.8% referred to the maximum likelihood estimation. This diagnostic performance was consistently observed in both independent case–control populations, with Center I achieving an AUC of 0.73 (sensitivity 81.3%, specificity 59.8%) and Center II an AUC of 0.76 (sensitivity 67.5%, specificity 76.7%) (Figure 1).

**Figure 2:**
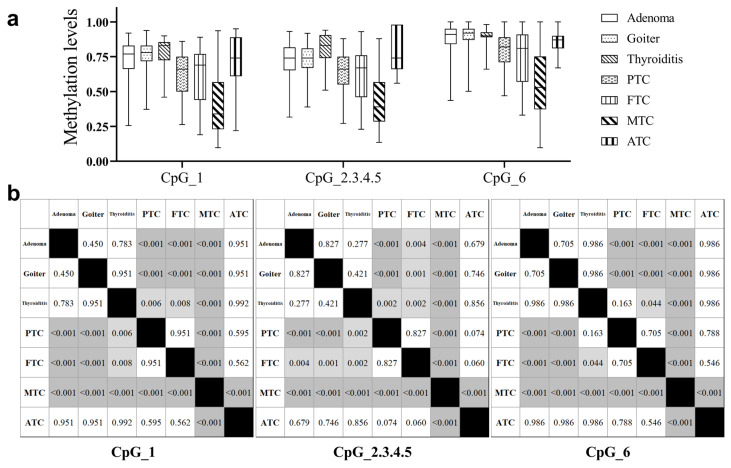
Comparison of *BCAR3* methylation across thyroid tumor subtypes. (**a**) Boxplot visualization of *BCAR3* methylation levels across different subtypes. (**b**) Matrix of *p* values from pairwise subgroup comparisons (Kruskal–Wallis test with Dunn’s post hoc test). Significant differences are indicated by gray shading, where a darker shade represents a smaller *p* value. The shading key is: white (*p* ≥ 0.05), light gray (*p* < 0.05), and dark gray (*p* < 0.001). Abbreviations: PTC, papillary thyroid carcinoma; FTC, follicular thyroid carcinoma; MTC, medullary thyroid carcinoma; ATC, anaplastic thyroid carcinoma.

We further evaluated the correlation between *BCAR3* methylation levels and key clinical characteristics in TC patients. Hypomethylation of *BCAR3* was enhanced in TC with aggressive clinicopathological features. Lower methylation levels at CpG_1 and CpG_6 were observed in TCs with larger tumor sizes (T2–4) and with involved lymph nodes compared to the one with T1 tumors and without lymph nodes involvement (all *p* < 0.01, Table 3). Notably, the methylation levels of all six *BCAR3* CpG sites decreased significantly with increasing tumor stage (*p* ≤ 0.006), and showed the lowest methylation in Stage III-IV diseases (Table 3). Analysis of *BCAR3* methylation across thyroid tumor subtypes revealed distinct patterns. No significant differences were observed among the three benign subtypes (adenoma, goiter, and thyroiditis) or between any benign subtype and anaplastic thyroid cancer (ATC) in pairwise comparisons (all *p* > 0.05). Papillary (PTC) and follicular thyroid carcinomas (FTC) exhibited similar methylation levels (PTC vs. FTC, *p* > 0.05), but were both significantly low than BTNs (all *p* < 0.005). Notably, medullary thyroid carcinoma (MTC) showed the lowest methylation values among all subtypes, with a reduction of approximately 35% to 48% compared to PTC and FTC (all Bonferroni-adjusted *p* < 0.05; Figure 2, Table S6).

### 3.2. BCAR3 Highly Expressed in Cancerous Thyroid Tissues and Cells

**Figure 3:**
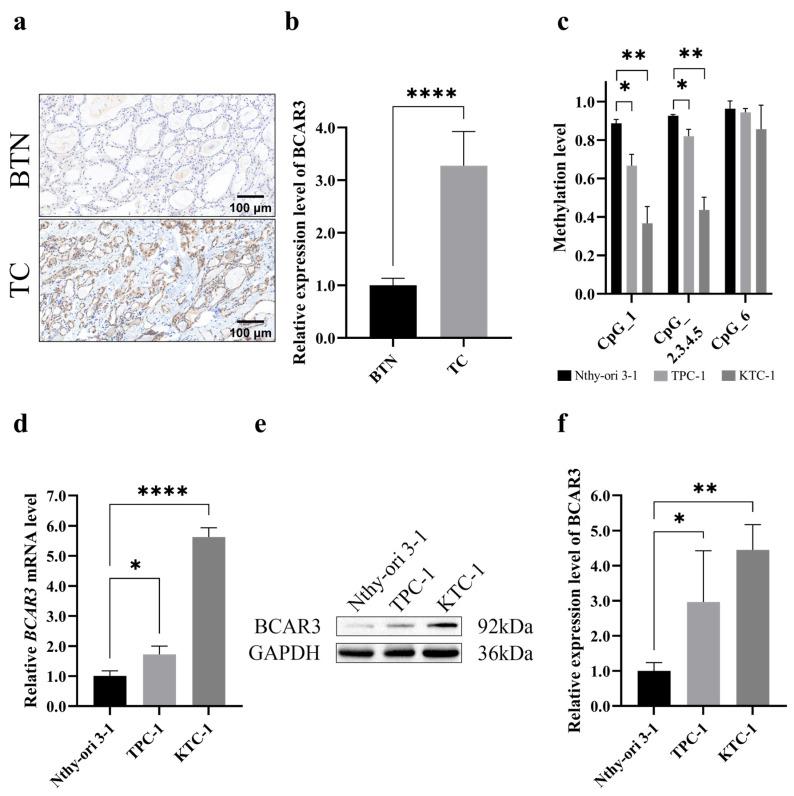
BCAR3 expression in benign and malignant thyroid tissues and three different cell lines (Nthy-ori 3-1, normal thyroid cell line; TPC-1, and KTC-1, thyroid cancer cell lines). (**a**,**b**) BCAR3 expression levels in BTN (n = 9) and TC (n = 9) tissues detected by immunohistochemistry (**a**). The bar graph quantifies the mean intensity of BCAR3 staining (**b**). Statistical significance was determined by an unpaired *t*-test. (**c**) *BCAR3* methylation levels of 3 different cell lines (n = 3 biological replicates), detected by MALDI-TOF mass spectrometry. Statistical comparisons of each cancer cell line (TPC-1, KTC-1) against the normal thyroid cell line (Nthy-ori 3-1) were performed using unpaired *t*-tests. (**d**) The relative mRNA levels of BCAR3 normalized to the internal control (GAPDH) detected by quantitative real-time PCR (n = 3). Statistical comparisons of each cancer cell line against the normal thyroid cell line were performed using unpaired *t*-tests. (**e**,**f**) BCAR3 protein expression detected by Western blot analysis, with GAPDH as a loading control (**e**). The quantitative assessment of protein expression levels is presented as the relative intensity of each protein band (n = 3). Statistical comparisons of each cancer cell line against the normal thyroid cell line were performed using unpaired *t*-tests (**f**). Data in all bar graphs are presented as mean with SD. * *p* value < 0.05; ** *p* value < 0.01; **** *p* value < 0.001. Abbreviations: TC, thyroid cancer; BTN, benign thyroid nodule. The uncropped blots are shown in Figure S7.

Next, we examined the expression of BCAR3 in TC using thyroid tissue sections from TC cases and BTN subject. Compared to BTN, TC tissues exhibited significantly stronger immunohistochemical staining for BCAR3 (*p* < 0.001; Figure 3a,b). *BCAR3* methylation levels in cell lines mirrored the results of that in tissues: five of the six CpG sites at *BCAR3* promotor region hypomethylated in two TC cell lines (TPC-1 and KTC-1) compared to the normal thyroid cell line (Nthy-ori 3-1) (*p* < 0.05 for CpG_1 and CpG_2.3.4.5; Figure 3c). Consistently, quantitative PCR revealed a significant upregulation of *BCAR3* mRNA levels in two TC cell lines compared to the normal thyroid cell line (*p* < 0.05; Figure 3d), while Western blot analysis confirmed a marked increase in BCAR3 protein expression in two TC cell lines compared to normal thyroid cell line (*p* < 0.05; Figure 3e,f).

### 3.3. Downregulation of BCAR3 Suppressed the Proliferation, Invasion and Migration of Thyroid Cancerous Cells via Regulating AKT/mTOR Pathway

**Figure 4:**
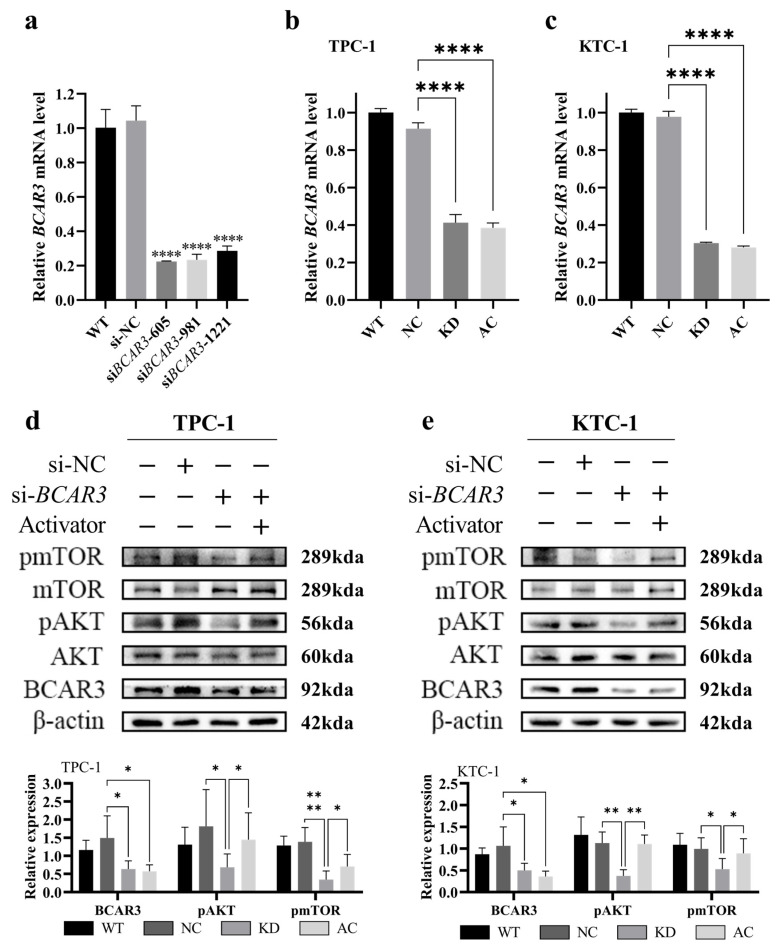
RNA interference target screening and *BCAR3* knockdown effect in TPC-1 and KTC-1 cells. The abbreviations of the four groups in different cells are as follows: WT, wild type; NC, negative control with si-NC treated; KD, knockdown by si-*BCAR3*; AC, knocked down and treated by PI3K activator. (**a**) The selection of *BCAR3* siRNA sequence. *BCAR3* mRNA expression in KTC-1 cells after transfection with three different siRNAs (si*BCAR3*-605, si*BCAR3*-981 and si*BCAR3*-1221) detected by quantitative real-time PCR. Statistical comparisons between each siRNA group and the negative control (si-NC) group were performed using unpaired *t*-tests. (**b**,**c**) Validation of *BCAR3* knockdown efficiency. *BCAR3* mRNA expression in TPC-1 (**b**) and KTC-1 (**c**) cells after siRNA-mediated knockdown detected by quantitative real-time PCR (n = 3). GAPDH was used as an internal control. Statistical significance was determined by an unpaired *t*-test. (**d**,**e**) Expression of phosphorylated mTOR (pmTOR), total mTOR, phosphorylated AKT (pAKT), total AKT, BCAR3 in TPC-1 (**d**) and KTC-1 (**e**) cells was detected by Western blot. β-actin was used as a loading control. The corresponding quantitative analysis of protein expression were shown in the bar graphs below. Statistical comparisons between specified groups (KD vs. NC; AC vs. KD) for each protein were performed using unpaired *t*-tests. Data in all bar graphs are presented as mean with SD; * *p* < 0.05, ** *p* < 0.01, **** *p* < 0.001. The uncropped blots are shown in Figures S8 and S9.

We next aimed to investigate the role of upregulated BCAR3 in TC development by knocking down *BCAR3* in cell lines using siRNA. The knockdown efficiency was examined by quantitative real-time PCR. As shown in Figure 4a, three siRNA sequence (si*BCAR3*-605, siB*CAR3*-981 and si*BCAR3*-1221) showed efficient knock down of *BCAR3* in KTC-1 cell line, and the best performed si*BCAR3*-605 was selected for subsequent studies.

To explore the potential function of BCAR3 and its role in the PI3K/AKT signaling pathway in TC, we conducted experiments involving *BCAR3* knockdown alone (KD group) and in combination with 1,3-dicaffeoylquinic acid, a PI3K activator (AC group). *BCAR3* was successfully downregulated in both TPC-1 and KTC-1 cell lines across the KD and AC groups when compared to the negative control (NC groups) (*p* < 0.001, KD vs. NC, AC vs. KD; Figure 4b,c). Western blot analysis indicated that *BCAR3* knockdown significantly reduced the phosphorylation of AKT and mTOR in both cell lines (*p* < 0.05, KD vs. NC; Figure 4d,e).

**Figure 5:**
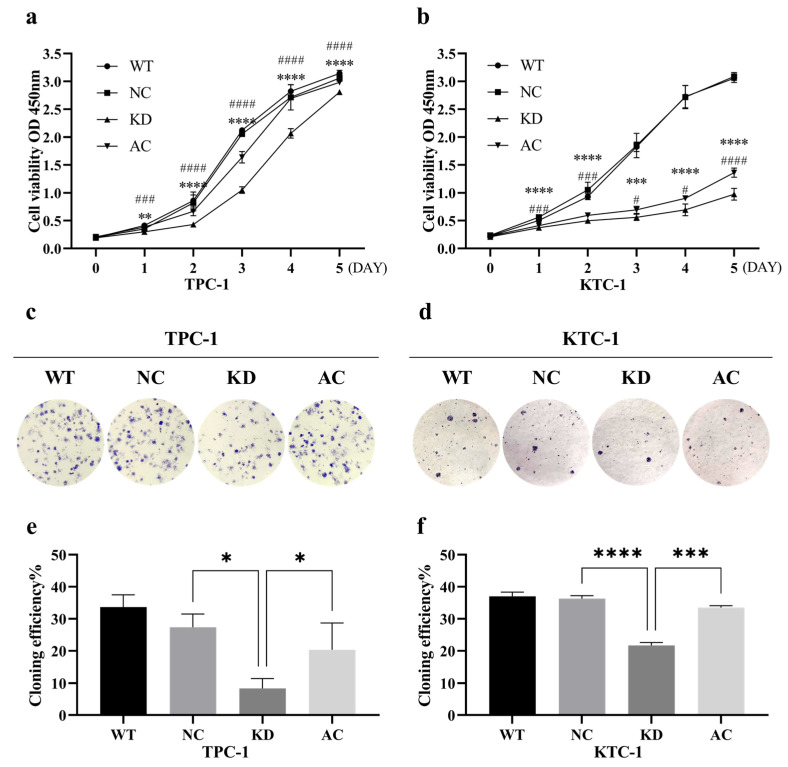
Effects of *BCAR3* knockdown and PI3K activation on cell proliferation and colony formation in thyroid cancer cell lines. The abbreviations of the four groups in different cells are as follows: WT, wild type; NC, negative control with si-NC treated; KD, knockdown by si-*BCAR3*; AC, knocked down and treated by PI3K activator. (**a**,**b**) Proliferation of TPC-1 (**a**) and KTC-1 (**b**) cells was measured using CCK-8 assay (n = 6 biological replicates). Data are presented as mean ± SD. Statistical comparisons between KD vs. NC, and AC vs. KD groups were assessed using unpaired *t*-tests ** *p* < 0.01, *** *p* < 0.005, **** *p* < 0.001, KD vs. NC; # *p* < 0.05, ### *p* < 0.005, #### *p* < 0.001, AC vs. KD. (**c**,**d**) Representative images of colony formation assays in TPC-1 (**c**) and KTC-1 (**d**) cells under the same four conditions (n = 3 biological replicates). (**e**,**f**) Quantification of colony formation efficiency in TPC-1 (**e**) and KTC-1 (**f**) cells. Data are presented as mean with SD. Statistical comparisons between KD vs. NC and AC vs. KD groups were performed using unpaired *t*-tests. * *p* < 0.05, *** *p* < 0.005, **** *p* < 0.001.

**Figure 6:**
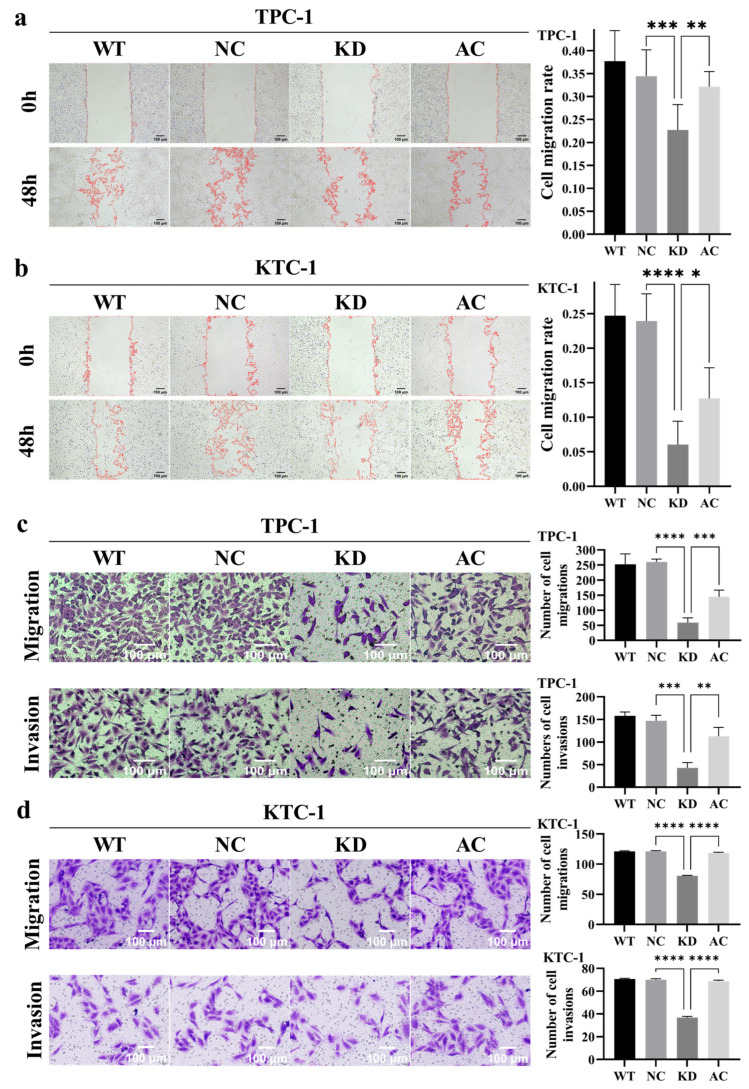
Effects of *BCAR3* knockdown and PI3K activation on cell migration and invasion in TPC-1 and KTC-1 thyroid cancer cell lines. The four experimental groups were: WT, wild type; NC, negative control with si-NC treated; KD, knockdown by si-*BCAR3*; AC, knocked down and treated by PI3K activator. (**a**,**b**) Cell migration in TPC-1 (**a**) and KTC-1 (**b**) cells was examined by wound healing assay. Quantitative analysis of the relative wound closure area is shown on the right (n = 6 biological replicates). Data are presented as mean with SD. (**c**,**d**) Cell migration and invasion in TPC-1 (**c**) and KTC-1 (**d**) cells was evaluated by transwell assay. Quantitative data on the number of migrated or invaded cells are shown on the right (n = 3 biological replicates). Data are presented as mean with SD. Statistical comparisons between KD vs. NC and AC vs. KD groups were performed using unpaired *t*-tests. * *p* < 0.05, ** *p* < 0.01, *** *p* < 0.005, **** *p* < 0.001.

Both the CCK-8 cell proliferation assay and the clonogenic assay revealed that silencing *BCAR3* significantly inhibited the proliferation in both TPC-1 and KTC-1 cell lines when compared to the NC groups (*p* < 0.05; Figure 5). Additionally, wound healing assays showed that the wound closure rate in the *BCAR3* KD group was significantly lower than that in the wild-type (WT) and NC groups in both cell lines (*p* < 0.05; Figure 6a,b). Moreover, *BCAR3* knockdown led to a significant reduction in both cell migration and invasion (*p* < 0.001; Figure 6c,d).

Furthermore, the activation of the PI3K/AKT pathway in *BCAR3* knockdown cells using 1,3-dicaffeoylquinic acid partially but significantly restored cell proliferation, migration, and invasion, thereby partially reversing the effects of *BCAR3* knockdown (*p* < 0.05 AC vs. KD; Figure 5 and Figure 6). However, the restored levels remained statistically lower than those in the WT and NC groups, indicating a partial functional rescue.

Importantly, the inhibitory effects on cell proliferation, migration, and invasion were consistently observed not only with the selected si*BCAR3*-605 but also with the two additional siRNAs (si*BCAR3*-981 and si*BCAR3*-1221), confirming the specificity of these phenotypes to *BCAR3* knockdown (Figures S4 and S5).

### 3.4. BCAR3 Promotes the Invasion of Thyroid Cancerous Cell Through EMT

**Figure 7:**
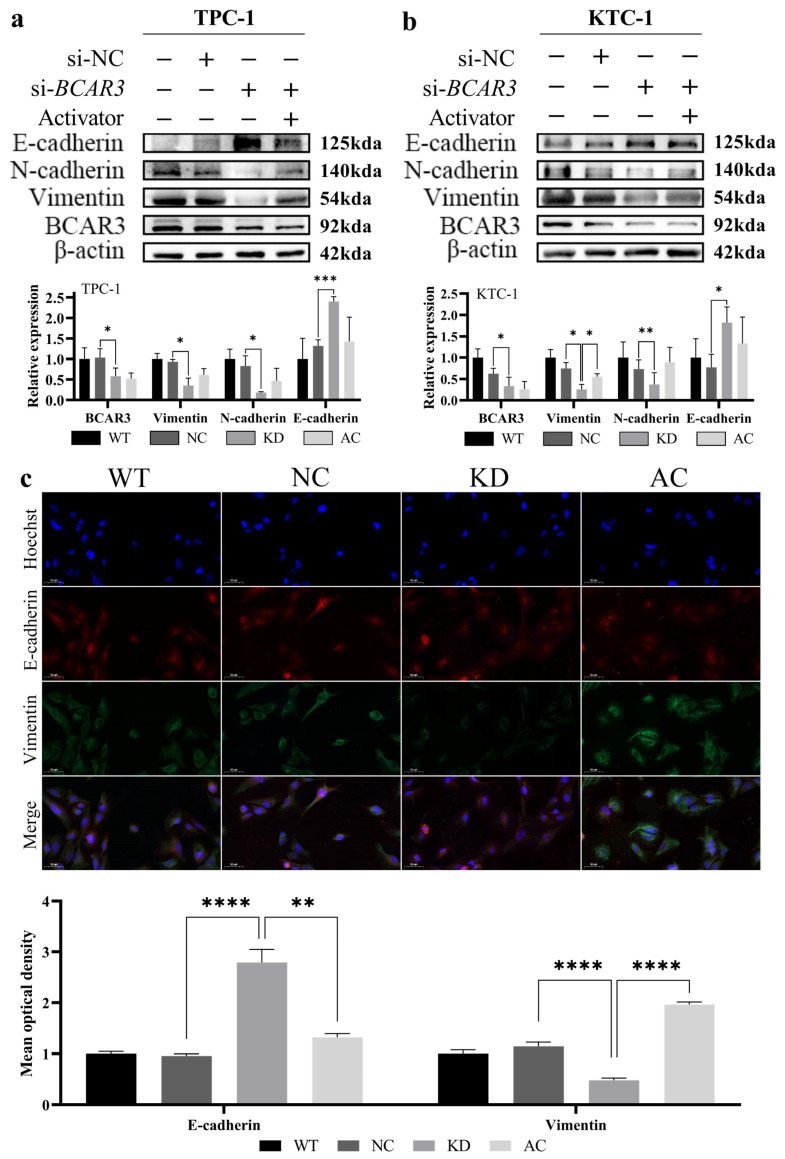
Effects of *BCAR3* knockdown on epithelial–mesenchymal transition (EMT) markers in TPC-1 and KTC-1 cells. The abbreviations of the four groups in different cells are as follows: WT, wild type; NC, negative control with si-NC treated; KD, knockdown by si-*BCAR3*; AC, knocked down and treated by PI3K activator. (**a**,**b**) Expression of BCAR3, E-cadherin, N-cadherin, and Vimentin in TPC-1 (**a**) and KTC-1 (**b**) cells across four conditions—wild-type (WT), negative control (NC), *BCAR3* knockdown (KD), and *BCAR3* knockdown with PI3K activator (AC)—was analyzed by Western blot. Quantification of relative protein band intensity is shown below the blots (n = 3 biological replicates). Data are presented as mean with SD. (**c**) The localization of E-cadherin (green) and Vimentin (red) in KTC-1 cells under the same conditions as in (**a**,**b**) was examined by immunofluorescence staining. Nuclei are stained with Hoechst (blue). The bar graph quantifies the mean fluorescence intensity of E-cadherin and Vimentin (n = 4 biological replicates). Data are presented as mean with SD. For all quantitative analyses, statistical comparisons between specific groups (KD vs. NC; AC vs. KD) were performed using unpaired *t*-tests. * *p* < 0.05, ** *p* < 0.01, *** *p* < 0.005, **** *p* < 0.001. The uncropped blots are shown in Figures S10 and S11.

We subsequently investigate the involvement of EMT, a pivotal mechanism in cancer metastasis, in the regulation of invasion and migration by BCAR3 in TC. Western blot analysis of EMT markers revealed that silencing BCAR3 significantly elevated E-cadherin expression while reducing levels of Vimentin and N-cadherin in both TPC-1 and KTC-1 cell lines (*p* < 0.05, KD vs. NC; Figure 7a,b). To further validate BCAR3’s role in EMT, we performed immunofluorescence staining in KTC-1 cells, which confirmed a marked increase in E-cadherin and a concurrent decrease in Vimentin upon *BCAR3* knockdown (*p* < 0.001, KD vs. NC; Figure 7c). These findings indicate that *BCAR3* knockdown altered the expression of EMT markers, which may be linked to its effects on cell proliferation, migration, and invasion. Moreover, activation of the PI3K/AKT pathway via 1,3-dicaffeoylquinic acid significantly attenuated the impact of *BCAR3* knockdown on EMT markers, as evidenced by decreased E-cadherin and increased Vimentin expression in the AC group compared to the KD group (*p* < 0.001, AC vs. KD; Figure 7). Notably, the expression levels of these markers in the AC group were intermediate between the KD and NC groups, consistent with a partial reversal of the EMT phenotype.

## 4. Discussion

The increasing detection of thyroid nodules and the overlap in clinical characteristics between benign and malignant nodules underscore the need for efficient, objective molecular markers to improve diagnosis and management [35]. Altered DNA methylation represents a significant event in cancer development and progression [16]. The straightforward detection of tissue DNA methylation levels in pathological biopsy samples at a low cost makes it feasible for DNA methylation to be used in clinical diagnosis or as a companion diagnostic tool [36,37]. In this study, we identified significant hypomethylation at six CpG sites in the 5′-UTR region of the *BCAR3* gene in TC samples compared to BTN samples. Logistic regression analysis further indicated that *BCAR3* hypomethylation is associated with an increased risk of TC. Although CpG_1 of this *BCAR3* amplicon is overlapped with rs11164983 (G-A), it presented similar OR comparing the TCs and BTNs as the other 5 CpG sites, suggesting the frequency of this SNP may have no significant difference between TC and BTN groups. Nevertheless, future studies should be cautions to the potential influence of SNPs via either avoiding SNP overlapped CpG sites or examining the frequency of SNPs in parallel. We also investigated the methylation status of the *BCAR3* in the Illumina 450K array based Cancer Genome Atlas (TCGA) thyroid cancer (THCA) dataset, and found available data for only one CpG site (cg04258676) in the 1kb flanking region of the analyzed amplicon (Figure S6a). It is noteworthy that the slightly opposite hypermethylation of cg04258676 in TC than the adjacent normal tissue (*p* = 0.083, Figure S6b) showed the regional specific DNA methylation regulation as has been reported in our previous studies [38,39,40], where different CpG sites/region within the same gene can present variant diseased related methylation patterns, and thus, could be moderated by distinct regulatory elements (e.g., enhancers, transcription factors) leading to divergent patterns in diseases [41]. Moreover, the observed correlation between progressive hypomethylation and aggressive clinicopathological features, including larger tumor size, lymph node metastasis, and advanced tumor stage, suggests that *BCAR3* may play a role in tumor aggressiveness. Differed from our previously reported lowest gene methylation in ATC [42], current study found the lowest *BCAR3* methylation detected in MTC. While subtyping PTC is relatively straightforward in surgical specimens, its accuracy is still limited when based solely on cytology [43,44]. Although the rare TC subtypes are limited at sample size, our study still highlighted the potential of methylation profiles may assist the traditional strategy to improve the diagnosis and classification of variant TC subtypes. Since PTC attribute to more than 90% of TCs, future multi-center collaborations are needed to accumulate larger series for the investigation and validation of subtype-specific methylation as well as its clinical application. Meanwhile, the TC and subtype specific methylation could provide novel insight into the initiation and progression of cancer, the biological mechanism for these methylation genes or epigenetic driving force would be meaningful to explore. Collectively, our results suggest that *BCAR3* hypomethylation may serve as a diagnostic biomarker, though further validation in larger, prospective cohorts is necessary for potential translational applications. Future studies combining the overexpression of *BCAR3* as well as multiple markers may further improve diagnostic accuracy.

The regulation of gene expression by DNA methylation is well-established [45]. In our study, we suggest that the upregulation of *BCAR3* in TC may be regulated by epigenetic mechanisms, particularly promoter hypomethylation—a well-known process in oncogene activation [46,47]. Our methylation analysis showed hypomethylation in TC tissues, which was consistent with increased BCAR3 protein expression, as confirmed by immunohistochemistry. This is in line with findings in other malignancies, such as breast cancer and head and neck cancer, where BCAR3 has been implicated in tumor progression [27,48]. Notably, we observed that the changes in *BCAR3* methylation and protein expression at the tissue level were mirrored at the cellular level. This series of experiments supports the hypothesis that methylation-driven BCAR3 expression plays a key role in thyroid carcinogenesis. It is also important to note that no methylation editing experiments (e.g., using dCas9-DNMT3A or dCas9-TET1 tools) or luciferase reporter assays were performed in our study to prove the causally link between methylation to *BCAR3* and expression. Such experiments should be considered in the future for the better understanding of mechanism of methylation.

Our phenotypic studies further revealed that *BCAR3* knockdown in TPC-1 and KTC-1 cells significantly reduced cellular proliferation, migration, invasion, and colony formation, underscoring its critical role in promoting aggressive tumor behavior. Additionally, silencing *BCAR3* led to the downregulation of mesenchymal markers, such as Vimentin and N-cadherin, along with upregulating the epithelial marker E-cadherin, reinforcing BCAR3’s role as a key regulator of EMT in TC. EMT is a key process in tumor progression, enabling epithelial cells to acquire a mesenchymal phenotype that enhances migration, invasion, and resistance to therapy [49,50]. This process is observed in various cancers, including TC [51,52,53]. The reversal of EMT markers upon BCAR3 depletion suggests its involvement in modulating cytoskeletal dynamics and cell adhesion, thereby facilitating tumor cell invasion and potentially metastasis.

Beyond its role in EMT, BCAR3 promotes tumor progression through the activation of multiple signaling pathways. Structurally, BCAR3 contains an N-terminal SH2-like domain, allowing interactions with phosphorylated tyrosine residues, and a C-terminal RasGEF-like domain, which lacks classical GEF activity [24,26,54]. Instead, BCAR3 binds BCAR1 at the C-terminus, forming a complex that recruits and activates Src kinase, leading to BCAR1 phosphorylation and downstream signaling activation [30,54,55]. Our results showed that *BCAR3* knockdown reduced pAKT and pmTOR levels, suggesting that BCAR3 promotes PI3K/AKT/mTOR signaling. Given that Src activation is an upstream driver of AKT phosphorylation, the BCAR3-BCAR1-Src complex likely facilitates this pathway in TC. Due to the unavailability of fresh-frozen tissue for optimal phospho-protein analysis, our study is limited by the absence of pAKT and pmTOR level assessment in the patient cohorts. Such validation in tissue should be considered in future studies. Although the PI3K activator (1,3-dicaffeoylquinic acid) successfully rescued the phenotypic effects of *BCAR3* knockdown, supporting the involvement of the PI3K/AKT pathway, we note that the use of a single activator is a limitation. Future studies employing alternative or more specific PI3K/AKT pathway modulators would be valuable to further confirm the specificity of the observed effects and rule out potential off-target actions. It is also noteworthy that PI3K/AKT pathway activation only partially rescued the phenotypic consequences of *BCAR3* knockdown, suggesting the involvement of additional signaling mechanisms. As a multi-domain adaptor protein, *BCAR3* may engage other key signaling nodes beyond PI3K/AKT, such as the MAPK/ERK or TGF-β/SMAD pathways, which could collectively contribute to its oncogenic functions [21,56]. Furthermore, BCAR3 depletion reversed EMT in TC cells, highlighting the importance of Src/AKT/mTOR signaling in driving aggressive tumor phenotypes. These findings position *BCAR3* as a regulator of invasive capabilities and tumor progression in TC and a potential candidate for therapeutic targeting, a concept that will require future functional validation in vivo. Furthermore, while our data support *BCAR3*’s role in tumor progression, future gain-of-function experiments through *BCAR3* overexpression would be valuable to definitively establish causality and further strengthen our conclusions.

## 5. Conclusions

In conclusion, this study associates *BCAR3* hypomethylation with TC progression, potentially through its regulation of the AKT/mTOR pathway and EMT progress. *BCAR3* knockdown was found to suppress multiple malignant behaviors in vitro, highlighting its significant role in thyroid cancer pathogenesis. These findings suggest *BCAR3* methylation status as a potential diagnostic biomarker and indicate *BCAR3* as a possible therapeutic target worthy of further investigation.

## Figures and Tables

**Table 1:** Clinical characteristics of subjects.

Characteristic	Group	Center I		Center II	
		BTN (n = 169)	TC (n = 136)	BTN (n = 173)	TC (n = 233)
Age (Median (IQR), years)		53 (46–60)	50 (43–57)	50 (37–55)	50 (38–57)
Gender (n, (%))	Female	132 (78.11%)	104 (76.47%)	133 (76.88%)	176 (75.54%)
	Male	37 (21.89%)	32 (23.53%)	40 (23.12%)	57 (25.52%)
Tumor length (Median (IQR), cm)		2.50 (1.50–3.50)	1.00 (0.83–1.50)	2.50 (1.50–3.50)	1.50 (1.20–2.40)
Tumor size (n, (%))	T1	-	114 (83.82%)	-	156 (67.83%)
	T2–4	-	22 (16.18%)	-	74 (32.17%)
Lymph node involvement (n, (%))	pN0	-	72 (52.94%)	-	88 (40.18%)
	pN1	-	64 (47.06%)	-	131 (59.82%)
Metastasis (n, (%))	M0	-	136 (100.00%)	-	228 (99.56%)
	M1	-	0 (0.00%)	-	1 (0.44%)
Tumor stage (n, (%))	Stage I	-	115 (84.56%)	-	170 (74.24%)
	Stage II	-	18 (13.24%)	-	41 (17.90%)
	Stage III–IV	-	3 (2.20%)	-	18 (7.86%)
Subtype (n, (%))	Adenoma	87 (51.48%)	-	105 (60.69%)	-
	Goiter	79 (46.75%)	-	58 (33.53%)	-
	Thyroiditis	3 (1.77%)	-	10 (5.78%)	-
	PTC	-	126 (92.65%)	-	177 (75.97%)
	FTC	-	2 (1.47%)	-	25 (10.73%)
	MTC	-	7 (5.15%)	-	25 (10.73%)
	ATC	-	1 (0.73%)	-	6 (2.57%)

Abbreviation BTN, benign thyroid nodule; TC, thyroid cancer; IQR, interquartile range; PTC, papillary thyroid carcinoma; FTC, follicular thyroid carcinoma; MTC, medullary thyroid carcinoma; ATC, anaplastic thyroid carcinoma.

**Table 2:** Association between *BCAR3* methylation and TC.

CpG Sites	BTN Median (IQR)	TC Median (IQR)	OR (95%CI) per −10% Methylation	*p* Value ^#^	Adjust *p* Value ^$^
Center I (136 TC vs. 169 BTN)					
CpG_1	0.79 (0.70–0.83)	0.67 (0.49–0.76)	1.53 (1.31–1.79)	**<0.001**	**<0.001**
CpG_2.3.4.5	0.76 (0.69–0.83)	0.67 (0.55–0.77)	1.40 (1.19–1.64)	**<0.001**	**<0.001**
CpG_6	0.92 (0.85–0.95)	0.82 (0.71–0.90)	1.45 (1.22–1.73)	**<0.001**	**<0.001**
Center II (233 TC vs. 173 BTN)					
CpG_1	0.77 (0.70–0.83)	0.63 (0.43–0.74)	1.70 (1.47–1.96)	**<0.001**	**<0.001**
CpG_2.3.4.5	0.72 (0.65–0.81)	0.64 (0.47–0.74)	1.55 (1.34–1.78)	**<0.001**	**<0.001**
CpG_6	0.91 (0.85–0.95)	0.80 (0.67–0.89)	1.87 (1.55–2.25)	**<0.001**	**<0.001**

^#^ *p* values were calculated by logistic regression with adjustment for age and gender. Significant *p* values are in bold; ^$^ Adjusted *p* values were calculated by *p* values × 3 (Bonferroni Adjustment). Abbreviation: BTN, benign thyroid nodule; TC, thyroid cancer; IQR, Interquartile Range.

**Table 3:** Correlation between *BCAR3* methylation and the clinical characteristics of thyroid cancers.

Clinical Characteristics	Group (n)	Median of Methylation Levels (IQR)		
		*BCAR3*_CpG_1	*BCAR3*_CpG_2.3.4.5	*BCAR3*_CpG_6
Tumor size	T1 (304)	0.66 (0.49–0.75)	0.66 (0.54–0.74)	0.83 (0.70–0.89)
	T2–4 (115)	0.57 (0.36–0.73)	0.58 (0.41–0.75)	0.76 (0.54–0.88)
	*p* value ^a^	**0.008**	**0.044**	**0.001**
Lymph node involvement	pN0 (176)	0.67 (0.47–0.78)	0.67 (0.51–0.76)	0.83 (0.68–0.90)
	pN1 (227)	0.61 (0.43–0.73)	0.62 (0.48–0.73)	0.77 (0.67–0.88)
	*p* value ^a^	**0.042**	0.061	**0.044**
Tumor stage	Stage I (322)	0.66 (0.49–0.75)	0.66 (0.53–0.74)	0.83 (0.71–0.89)
	Stage II (67)	0.56 (0.37–0.75)	0.61 (0.42–0.78)	0.73 (0.56–0.89)
	Stage III–IV (29)	0.36 (0.27–0.71)	0.45 (0.36–0.71)	0.60 (0.42–0.83)
	*p* value ^b^	**0.001**	**0.006**	**<0.001**

^a^ *p* values were calculated by Mann–Whitney U test. ^b^ *p* values were calculated by Kruskal–Wallis test. Significant *p* values are in bold. Abbreviation: IQR, interquartile range.

## Data Availability

The original contributions presented in this study are included in the article/Supplementary Material. Further inquiries can be directed to the corresponding author.

## Supplementary Materials

The following supporting information can be downloaded at https://www.mdpi.com/article/10.3390/cancers18020267/s1, Figure S1: DNA methylation and relative expression of *BCAR3* in thyroid nodules; Figure S2: Schematic of the *BCAR3* amplicon for methylation analysis; Figure S3: Replication of methylation levels at CpG site cg10546447 (*BCAR3*) by two independent methods; Figure S4: Effects of *BCAR3* knockdown by 2 additional siRNAs on cell proliferation and colony formation in thyroid cancer cell lines; Figure S5: Effects of BCAR3 knockdown by 2 additional siRNAs on cell migration and invasion in TPC-1 and KTC-1 thyroid cancer cell lines; Figure S6: *BCAR3* CpG sites in the TCGA-THCA cohort; Figure S7: The original Western blotting images of Figure 3e, evaluating BCAR3 in Nthy-ori 3-1, TPC-1 and KTC-1 cells; Figure S8: The original Western blotting images of Figure 4d, evaluating BCAR3, akt, pakt, mtor, and pmtor in TPC-1 cells of 4 groups; Figure S9: The original Western blotting images of Figure 4e, evaluating BCAR3, akt, pakt, mtor, and pmtor in KTC-1 cells of 4 groups; Figure S10: The original Western blotting images of Figure 7a, evaluating BCAR3, Vimentin, E-cadherin, and N-cadherin in TPC-1 cells of 4 groups; Figure S11: The original Western blotting images of Figure 7b, evaluating BCAR3, Vimentin, E-cadherin, and N-cadherin in KTC-1 cells of 4 groups; Table S1: Clinical characteristics of subjects; Table S2: The bisulfite-specific primers and the amplicon sequences for *BCAR3* gene; Table S3: siRNA sequences; Table S4: Primers for qPCR; Table S5: Association between *BCAR3* methylation and TC; Table S6: Methylation levels of different subtypes of thyroid tumor.

## Abbreviations

The following abbreviations are used in this manuscript:

TC	thyroid cancer
OR	odds ratio
EMT	epithelial-to-mesenchymal transition
FNAB	fine-needle aspiration biopsy
AJCC	American Joint Committee on Cancer
FFPE	formalin-fixed paraffin-embedded
BTN	benign thyroid nodule
IQR	interquartile range
PTC	papillary thyroid carcinoma
FTC	follicular thyroid carcinoma
MTC	medullary thyroid carcinoma
ATC	anaplastic thyroid carcinoma
MALDI-TOF	matrix-assisted laser desorption/ionization–time of flight
IHC	immunohistochemistry
IF	immunofluorescence staining
