# Supplementary material for: BCAR3 Hypomethylation as a Potential Diagnostic Marker for Thyroid Cancer and Its Mechanism via Promoting EMT and AKT/mTOR Pathway
Source: Cancers (Basel). 2026 Jan 15;18(2):267. doi: 10.3390/cancers18020267 (PMC12839260; doi:10.3390/cancers18020267)
Supplement: Supplementary file 1 [file cancers-18-00267-s001.zip › cancers-3955090-supplementary.pdf]

Table S1. Clinical characteristics of subjects.

Characteristic	Group	BTN (n=371)	TC (n=422)
Age (Median (IQR), years)		51 (42-58)	50 (40-57)
Gender (n, (%))	Female	287 (77.36%)	317 (75.12%)
	Male	84 (22.64%)	105 (24.88%)
Tumor length (Median (IQR), cm)		2.50 (1.50-3.50)	1.50 (1.00-2.00)
Tumor size (n, (%))	T1	-	304 (72.55%)
	T2-4	-	115 (27.45%)
Lymph node involvement (n, (%))	pN0	-	176 (43.67%)
	pN1	-	227 (56.33%)
Metastasis (n, (%))	M0	-	416 (99.76%)
	M1	-	1 (0.24%)
Tumor stage (n, (%))	Stage I	-	322 (77.03%)
	Stage II	-	67 (16.03%)
	Stage III-IV	-	29 (6.94%)
Subtype (n, (%))	Adenoma	192 (51.75%)	-
	Goiter	166 (44.74%)	-
	Thyroiditis	13 (3.51%)	-
	PTC	-	332 (78.67%)
	FTC	-	39 (9.24%)
	MTC	-	44 (10.43%)
	ATC	-	7 (1.66%)

Abbreviation BTN, benign thyroid nodule; TC, thyroid cancer; IQR, interquartile range; PTC, papillary thyroid carcinoma; FTC, follicular thyroid carcinoma; MTC, medullary thyroid carcinoma; ATC, anaplastic thyroid carcinoma.

Table S2. The bisulfite-specific primers and the amplicon sequences for *BCAR3* gene.

Forward	5′- aggaagagagGGATAGGAAGTGTTTTATGATATGAAG -3′
Reverse	5′- cagtaatacgactcactatagggagaaggctCACCAAAAACCAAACATACTCCTAA -3′
Amplicon	GGACAGGAAGTGCCCTATGATATGAAGCAAGCACACAACAAACCTCCT<br>GGACCTGTCCGCATCACACTGTGACTCACCAGCCGCGCGCGTGCTGGGC<br>AGGTAGAGGCTGGAGCGGTCAGGAGCATGCCTGGCTCCTGGTG

Uppercase letters indicate the sequence-specific primer regions, and nonspecific tags are shown in lowercase letters. The measurable CpG sites in the amplicon were underlined.

Table S3. siRNA sequences.

BCAR3-1221-human	Forward	5′-GCAAGGUGCCGUUCCUCAAGGTT-3′
	Reverse	5′-CCUUGAGGAACGGCACCUUGCTT-3′
BCAR3-981-human	Forward	5′-GAAGAGCCUUGUCCCUCAAAGTT-3′
	Reverse	5′-CUUUGAGGGACAAGGCUCUUCTT-3′
BCAR3-605-human	Forward	5′-CGGACAGUUCUGCGACUCAGCTT-3′
	Reverse	5′-GCUGAGUCGCAGAACUGUCCGTT-3′

Table S4. Primers for qPCR.

BCAR3 (Human)-RT-F	CAACTACTGTGAACTGAACC
BCAR3 (Human)-RT-R	ATTCTGCTTGGCTGTGAG
GAPDH (Human)-RT-F	GGAGCGAGATCCCTCCAAAAT
GAPDH (Human)-RT-R	GGCTGTTGTCATACTTCTCATGG

Table S5. Association between *BCAR3* methylation and TC

CpG sites	BTN median (IQR)	TC median (IQR)	OR (95%CI) per -10% methylation	*P* **value**#	*adj.P* **value**$
Center I (136 TC vs. 169 BTN)					
CpG_1	0.79 (0.70-0.83)	0.67 (0.49-0.76)	1.53 (1.31-1.79)	< 0.001	< 0.001
CpG_2.3.4.5	0.76 (0.69-0.83)	0.67 (0.55-0.77)	1.40 (1.19-1.64)	< 0.001	< 0.001
CpG_6	0.92 (0.85-0.95)	0.82 (0.71-0.90)	1.45 (1.22-1.73)	< 0.001	< 0.001
Center Ⅱ (233 TC vs. 173 BTN)					
CpG_1	0.77 (0.70-0.83)	0.63 (0.43-0.74)	1.70 (1.47-1.96)	< 0.001	< 0.001
CpG_2.3.4.5	0.72 (0.65-0.81)	0.64 (0.47-0.74)	1.55 (1.34-1.78)	< 0.001	< 0.001
CpG_6	0.91 (0.85-0.95)	0.80 (0.67-0.89)	1.87 (1.55-2.25)	< 0.001	< 0.001

#*P* values were calculated by logistic regression with adjustment for age and gender. Significant *P* values are in bold; $Adjusted *P* values were calculated by *P* values*3 (Bonferroni Adjustment). Abbreviation: BTN, benign thyroid nodule; TC, thyroid cancer; IQR, Interquartile Range.

Table S6. Methylation levels of different subtypes of thyroid tumor

Subtype(n)	Median of Methylation Levels (IQR)		
	*BCAR3*_CpG_1	*BCAR3*_CpG_2.3.4.5	*BCAR3*_CpG_6
Adenoma (192)	0.77 (0.66-0.83)	0.74 (0.65-0.82)	0.91 (0.84-0.95)
Goiter (166)	0.78 (0.72-0.83)	0.74 (0.67-0.81)	0.92 (0.87-0.95)
Thyroiditis (13)	0.83 (0.73-0.86)	0.83 (0.74-0.91)	0.90 (0.89-0.93)
PTC (332)	0.66 (0.50-0.75)	0.66 (0.55-0.75)	0.82 (0.71-0.89)
FTC (39)	0.69 (0.44-0.77)	0.67 (0.46-0.76)	0.81 (0.57-0.91)
MTC (44)	0.34 (0.23-0.57)	0.39 (0.29-0.57)	0.53 (0.37-0.75)
ATC (7)	0.74 (0.61-0.89)	0.74 (0.66-0.98)	0.87 (0.81-0.90)
P value[b]	<0.001	<0.001	<0.001
adj.P value[$]	<0.001	<0.001	<0.001

[b]*P* values were calculated by Kruskal Wallis test. [$]*P* values were adjusted by Bonferroni Adjustment. Significant *P* values are in bold.
Abbreviation: PTC, papillary thyroid carcinoma; FTC, follicular thyroid carcinoma; MTC, medullary thyroid carcinoma; ATC, Anaplastic thyroid cancer; IQR, interquartile range.

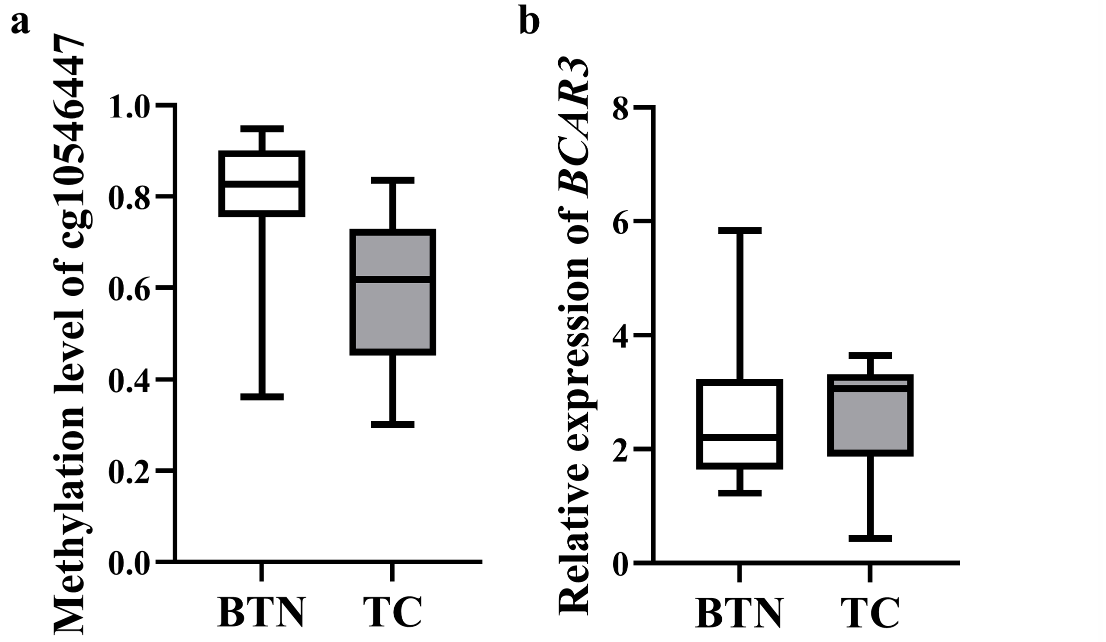

Figure S1. DNA methylation and relative expression of *BCAR3* in thyroid nodules. (a) Methylation level of the CpG site cg10546447 within the *BCAR3* gene, as determined by the Illumina 850K methylation array in 7 TC and 11 BTNs. (b) Relative mRNA expression of *BCAR3*, measured by RNA sequencing (RNA-seq) in 7 TC and 11 BTNs. Abbreviations: BTN: benign thyroid nodule; TC, thyroid cancer.

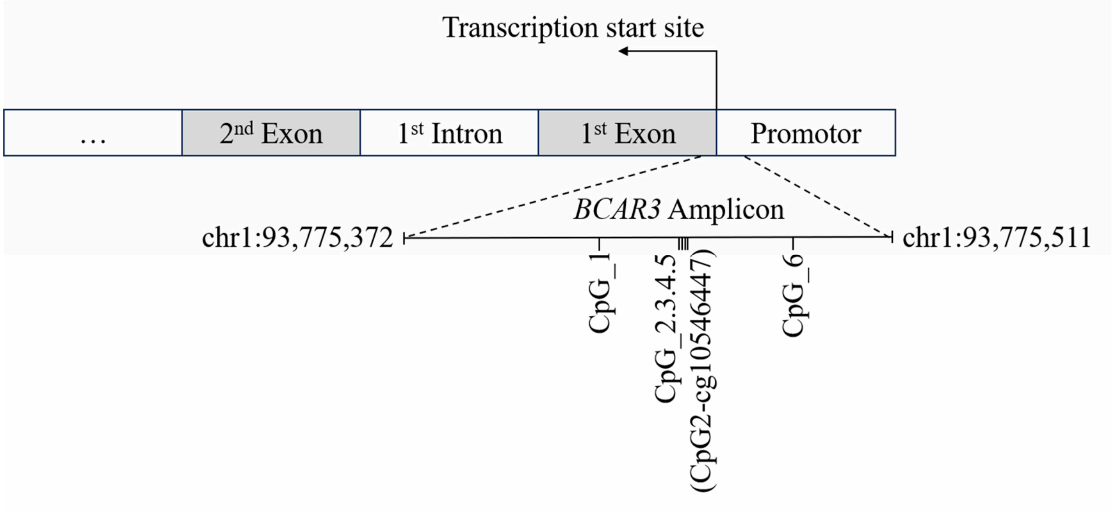

Figure S2. Schematic of the *BCAR3* amplicon for methylation analysis. Diagram shows the 140 bp target region (chr1:93,775,372-93,775,511, build GRCh38/hg38) located across the *BCAR3* promoter and 1st exon, analyzed by MALDI-TOF mass spectrometry. The locations of the measured six quantified CpG sites (CpG_1 to CpG_6) in the amplicon are presented, with CpG_2 corresponding to cg10546447.

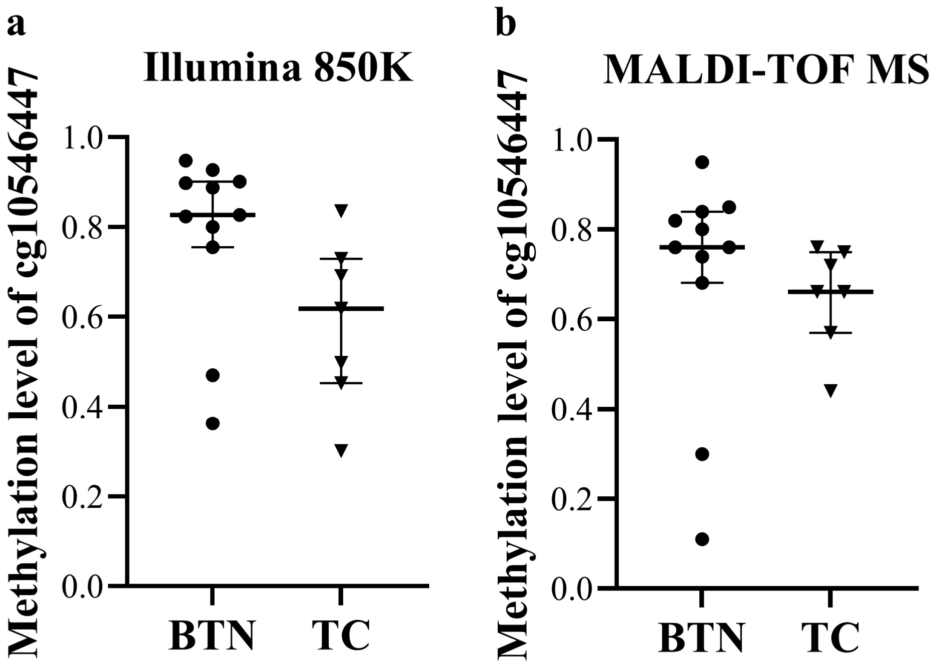

Figure S3. Replication of methylation levels at CpG site cg10546447 (*BCAR3*) by two techniques. (a) Methylation levels of cg10546447 measured by the Illumina 850K methylation array in 7 TC and 11 BTN samples. (b) Methylation levels of the same CpG site (cg10546447) analyzed by MALDI-TOF mass spectrometry (MS) in the same set of samples. Abbreviations: BTN: benign thyroid nodule; TC, thyroid cancer.

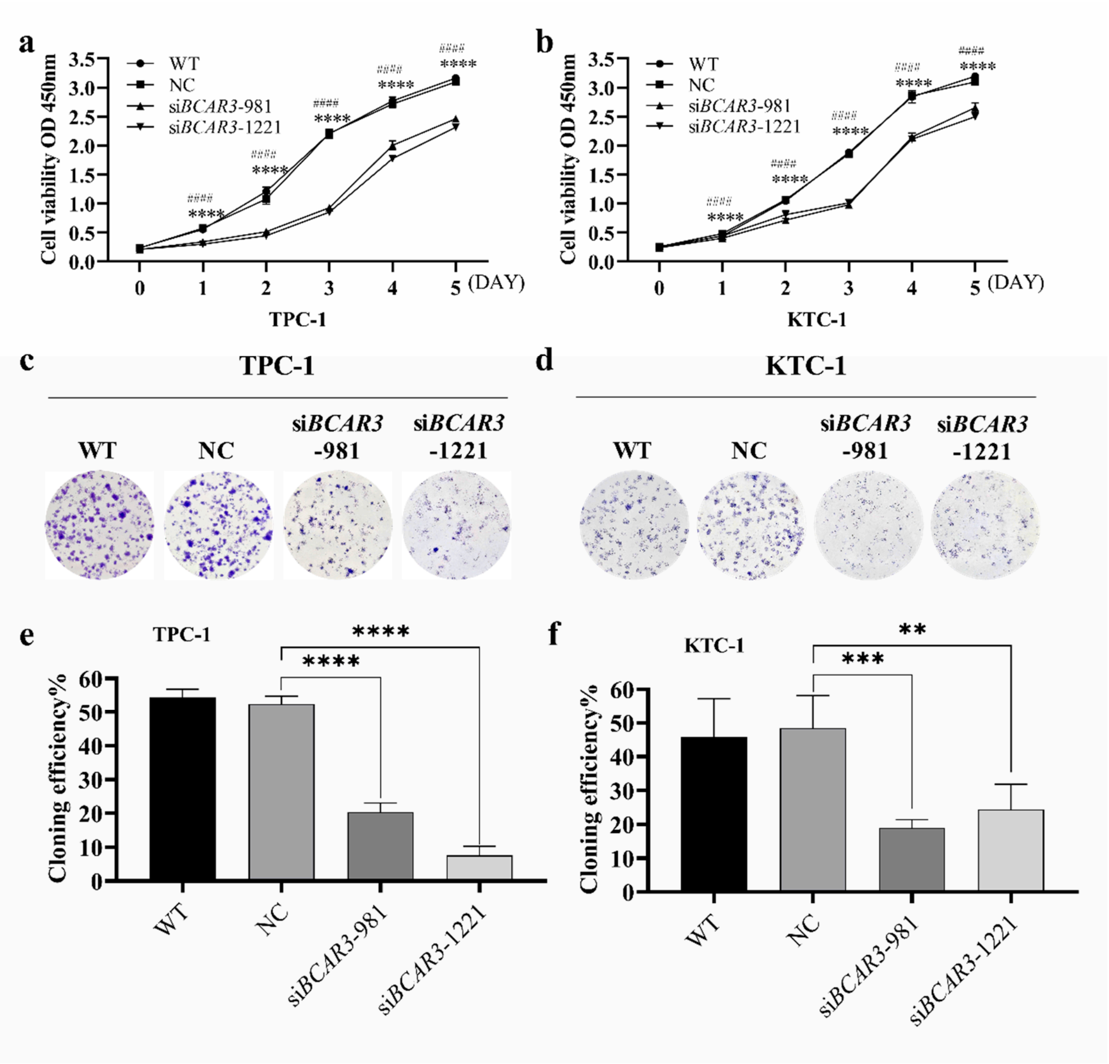

Figure S4. Effects of *BCAR3* knockdown by 2 additional siRNAs on cell proliferation and colony formation in thyroid cancer cell lines. The abbreviations of the four groups in different cells are as follows: WT, wild type; NC, negative control with si-NC treated; si*BCAR3*-981, knockdown by si*BCAR3*-981; si*BCAR3*-1221, knockdown by si*BCAR3*-1221. (a-b) Proliferation of TPC-1 (a) and KTC-1 (b) cells was measured using CCK-8 assay (n = 6 biological replicates). Data are presented as mean ± SD. Statistical comparisons between si*BCAR3*-981 vs. NC, and si*BCAR3*-1221 vs. NC groups were assessed using unpaired t-tests. *****P* < 0.001, si*BCAR3*-981 vs. NC; ###*P* < 0.005, ####*P* < 0.001, si*BCAR3*-1221 vs. NC. (c-d) Representative images of colony formation assays in TPC-1 (c) and KTC-1 (d) cells under the same four conditions (n = 3 biological replicates). (e-f) Quantification of colony formation efficiency in TPC-1 (e) and KTC-1 (f) cells. Data are presented as mean with SD. Statistical comparisons between si*BCAR3*-981 vs. NC and si*BCAR3*-1221 vs. NC groups were performed using unpaired t-tests. ***P* < 0.01, ****P* < 0.005, *****P* < 0.001.

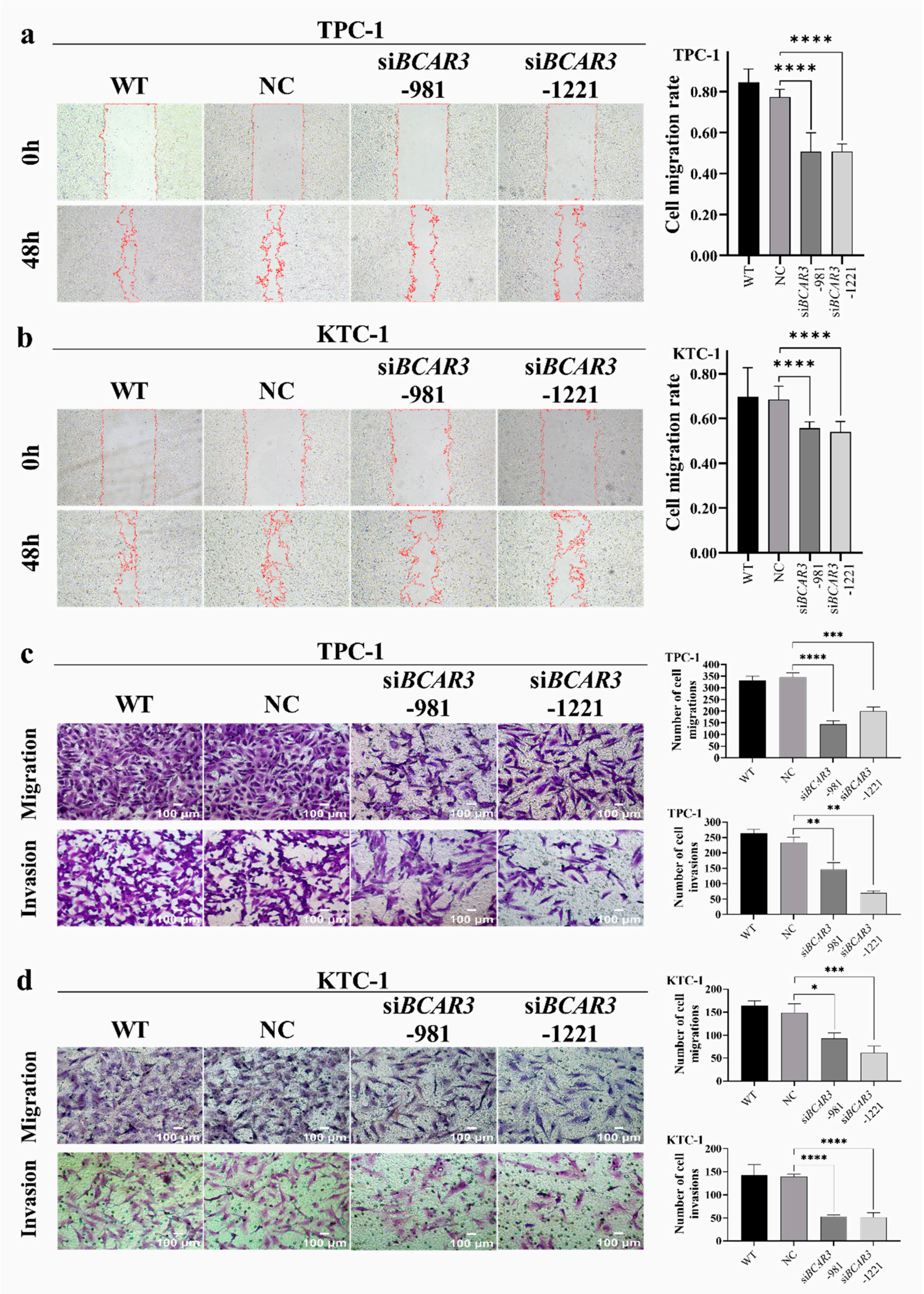

Figure S5. Effects of *BCAR3* knockdown by 2 additional siRNAs on cell migration and invasion in TPC-1 and KTC-1 thyroid cancer cell lines. The four experimental groups were: WT, wild type; NC, negative control with si-NC treated; si*BCAR3*-981, knockdown by si*BCAR3*-981; si*BCAR3*-1221, knockdown by si*BCAR3*-1221. (a-b) Cell migration in TPC-1 (a) and KTC-1 (b) cells was examined by wound healing assay. Quantitative analysis of the relative wound closure area is shown on the right (n = 6 biological replicates). Data are presented as mean with SD. (c-d) Cell migration and invasion in TPC-1 (c) and KTC-

1 (d) cells was evaluated by transwell assay. Quantitative data on the number of migrated or invaded cells are shown on the right (n = 3 biological replicates). Data are presented as mean with SD. Statistical comparisons between si*BCAR3*-981 vs. NC and si*BCAR3*-1221 vs. NC groups were performed using unpaired t-tests. *$P < 0.05$, **$P < 0.01$, ***$P < 0.005$, ****$P < 0.001$.

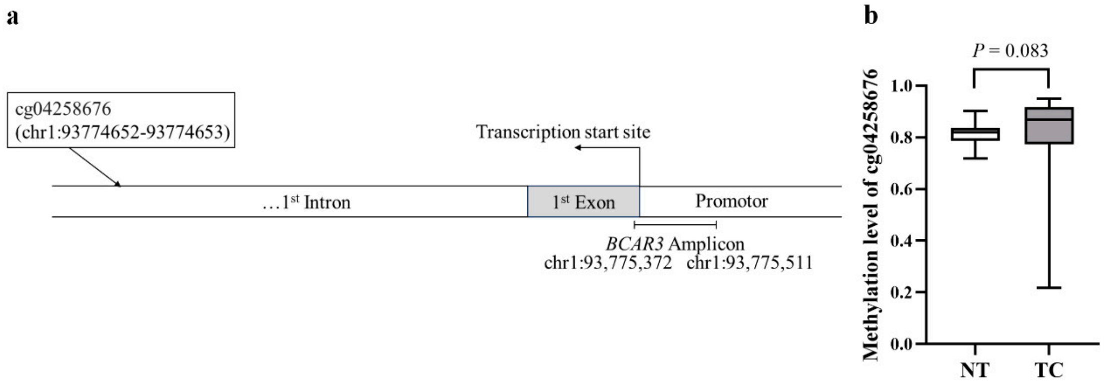

Figure S6. *BCAR3* CpG sites in the TCGA-THCA cohort. (a) In the Illumina 450K array based TCGA-THCA database, cg04258676 (719bp downstream) was the only interrogated CpG site in the 1kb flanking region of the analyzed amplicon. (b) Methylation level of cg04258676 in thyroid cancer (TC, n=505) and adjacent normal thyroid tissue (NT, n=50). The P value was calculated by unpaired t-test.

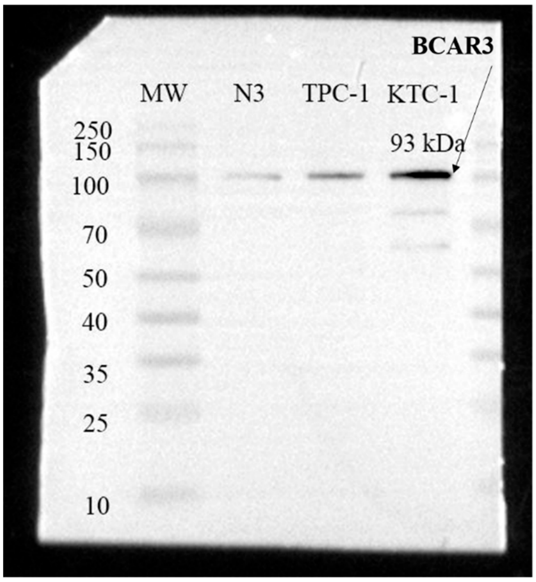

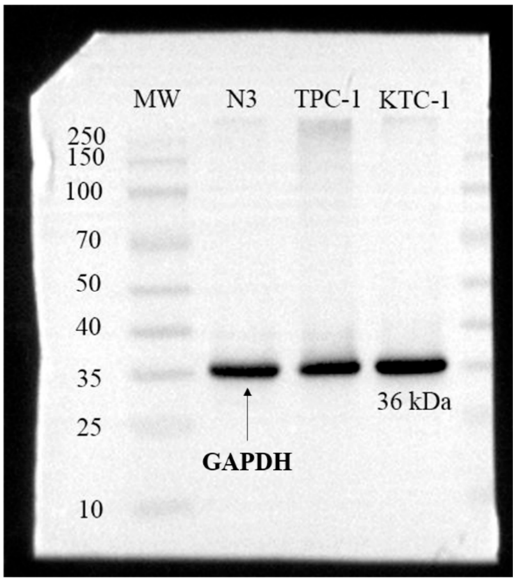

Figure S7. The original Western blotting images of of Figure 3e, evaluating BCAR3 in Nthy-ori 3-1, TPC-1 and KTC-1 cells.

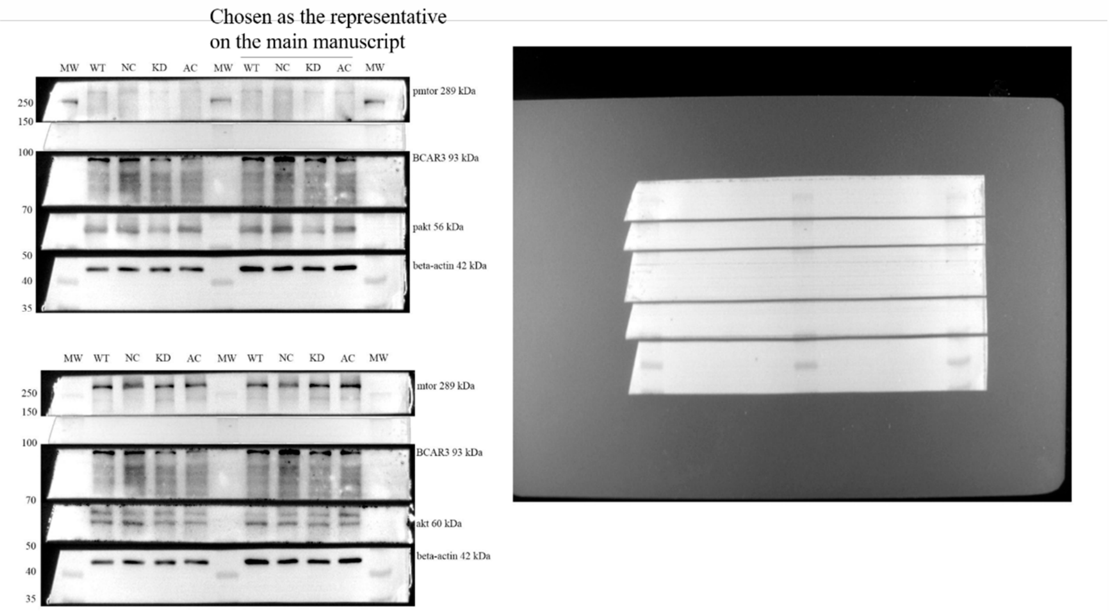

Figure S8. The original Western blotting images of Figure 4d, evaluating BCAR3, akt, pakt, mtor, and pmtor in TPC-1 cells of 4 groups. Abbreviations: NW, molecular weight; WT, wild type; NC: negative control; KD, knocked-down by si*BCAR3*-605; AC, Knocked-down and treated by PI3K activator.

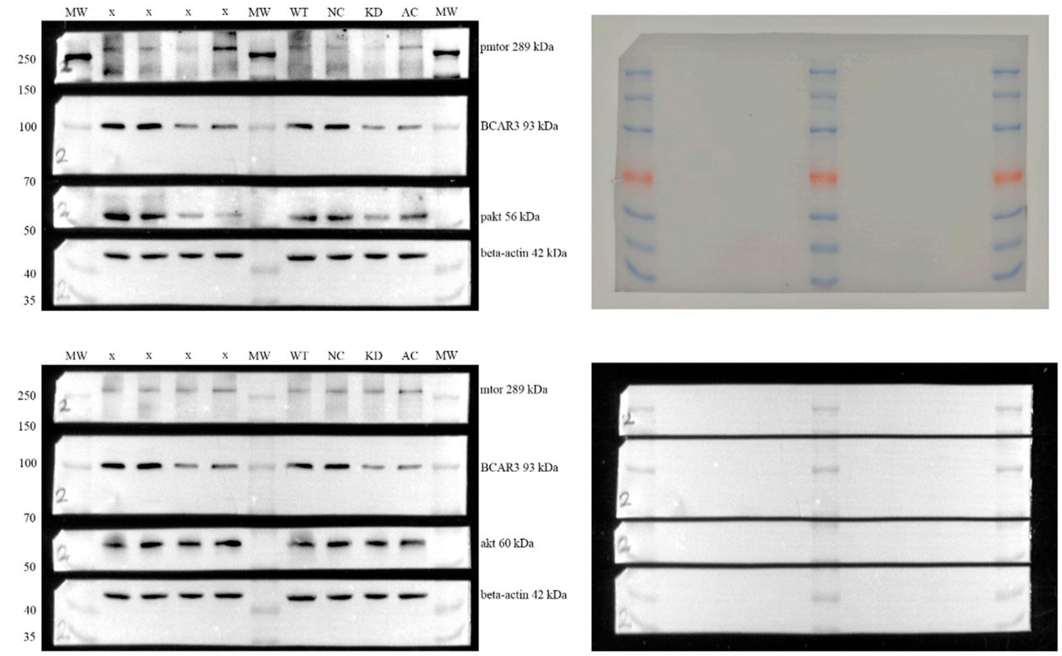

Figure S9. The original Western blotting images of Figure 4e, evaluating BCAR3, akt, pakt, mtor, and pmtor in KTC-1 cells of 4 groups. Abbreviations: NW, molecular weight; WT, wild type; NC: negative control; KD, knocked-down by si*BCAR3*-605; AC, Knocked-down and treated by PI3K activator. X: samples not included in the evaluation.

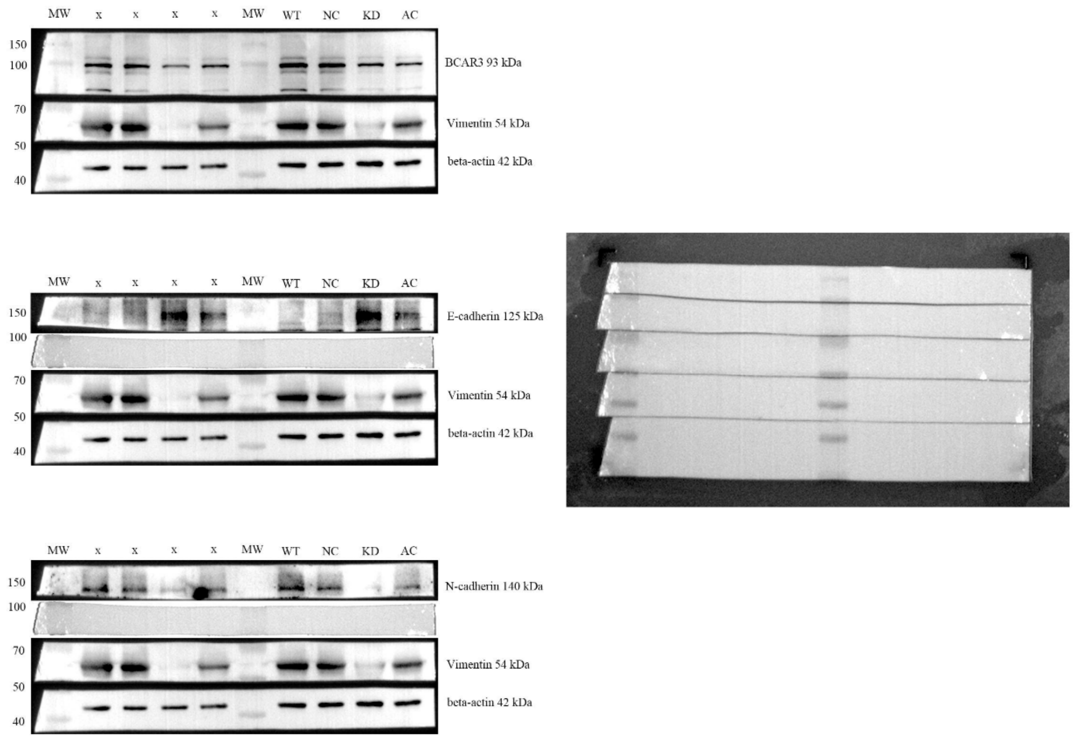

Figure S10. The original Western blotting images of Figure 7a, evaluating BCAR3, Vimentin, E-cadherin, and N-cadherin in TPC-1 cells of 4 groups. Abbreviations: NW, molecular weight; WT, wild type; NC: negative control; KD, knocked-down by si*BCAR3*-605; AC, Knocked-down and treated by PI3K activator. X: samples not included in the evaluation.

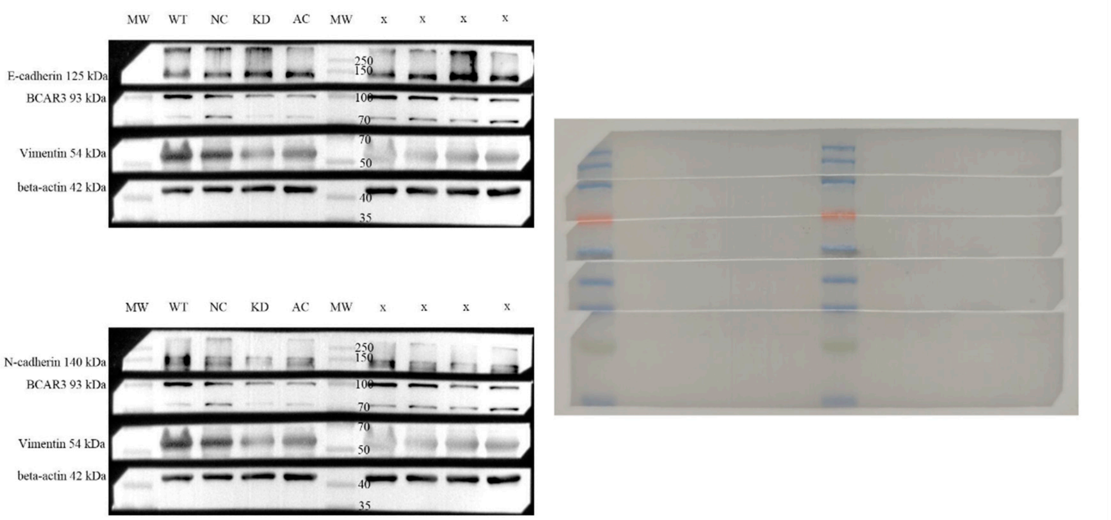

Figure S11: The original Western blotting images of Figure 7b, evaluating BCAR3, Vimentin, E-cadherin, and N-cadherin in KTC-1 cells of 4 groups. Abbreviations: NW, molecular weight; WT, wild type; NC: negative control; KD, knocked-down by si*BCAR3*-605; AC, Knocked-down and treated by PI3K activator. X: samples not included in the evaluation.
